# A review on the efficacy of artificial intelligence for managing anxiety disorders

**DOI:** 10.3389/frai.2024.1435895

**Published:** 2024-10-16

**Authors:** K. P. Das, P. Gavade

**Affiliations:** ^1^Department of Computer Science, Christ University, Bengaluru, India; ^2^Independent Practitioner, San Francisco, CA, United States

**Keywords:** artificial intelligence, GAD therapy, anxiety, mental health disorder, natural language proceeding (NLP)

## Abstract

Anxiety disorders are psychiatric conditions characterized by prolonged and generalized anxiety experienced by individuals in response to various events or situations. At present, anxiety disorders are regarded as the most widespread psychiatric disorders globally. Medication and different types of psychotherapies are employed as the primary therapeutic modalities in clinical practice for the treatment of anxiety disorders. However, combining these two approaches is known to yield more significant benefits than medication alone. Nevertheless, there is a lack of resources and a limited availability of psychotherapy options in underdeveloped areas. Psychotherapy methods encompass relaxation techniques, controlled breathing exercises, visualization exercises, controlled exposure exercises, and cognitive interventions such as challenging negative thoughts. These methods are vital in the treatment of anxiety disorders, but executing them proficiently can be demanding. Moreover, individuals with distinct anxiety disorders are prescribed medications that may cause withdrawal symptoms in some instances. Additionally, there is inadequate availability of face-to-face psychotherapy and a restricted capacity to predict and monitor the health, behavioral, and environmental aspects of individuals with anxiety disorders during the initial phases. In recent years, there has been notable progress in developing and utilizing artificial intelligence (AI) based applications and environments to improve the precision and sensitivity of diagnosing and treating various categories of anxiety disorders. As a result, this study aims to establish the efficacy of AI-enabled environments in addressing the existing challenges in managing anxiety disorders, reducing reliance on medication, and investigating the potential advantages, issues, and opportunities of integrating AI-assisted healthcare for anxiety disorders and enabling personalized therapy.

## 1 Introduction

Anxiety disorders are prevalent psychiatric conditions characterized by excessive and prolonged anxiety in response to various stimuli. The average lifetime prevalence of any anxiety disorder is approximately 16%, with a 12-month prevalence of around 11% (Kessler et al., 2009). These prevalence rates differ among studies, with higher estimates in developed Western countries compared to developing nations. Moreover, anxiety disorders are among the most prevalent mental health conditions, with a higher likelihood that the burden of these disorders will grow (Su et al., 2022). In recent years, a notable segment of the population, predominantly comprising the younger demographic, has suffered from mental health disorders, including anxiety and depression. Anxiety disorders, including conditions like depression, significantly impact medical care consumption. Failure to diagnose these conditions often leads to increased medical utilization as healthcare providers seek physical explanations for the symptoms.

Additionally, when depression co-exist with other general medical conditions, patient adherence to treatment worsens and reduces the chances of improvement or recovery from the other condition (Goldman et al., 1999). However, access to adequate treatment remains elusive to many within this cohort. This predicament reflects the broader societal context in which individuals facing mental health challenges hesitate to acknowledge their condition due to societal norms and pressure. Furthermore, for those who overcome societal stigmatization, the significant financial burden of seeking professional assistance poses an obstacle to accessibility (Furqan et al., 2020).

In a study exploring the prevalence of anxiety disorders, the authors analyzed the findings of a global survey conducted by the World Health Organization (WHO). The survey revealed that mental health conditions like depression, schizophrenia, and personality disorders are over-represented in inpatient treatment settings due to certain features of these disorders requiring hospitalization (Sartorius et al., 1993). In contrast, patients with anxiety disorders are generally underrepresented in inpatient care as their condition rarely necessitates hospitalization. These findings suggest a significant underestimation and inadequate treatment of anxiety disorders, indicating a need for increased awareness and appropriate interventions (Bandelow and Michaelis, 2015). Moreover, the standard anxiety disorder management guidelines across countries endorse psychotherapy as the primary therapeutic approach, including modalities such as cognitive behavioral therapy, relaxation therapy, exposure-response prevention, and several other psychotherapeutic interventions (Katzman et al., 2014; Bandelow et al., 2022). However, the limited availability of qualified therapists, especially in remote or developing areas, affects the accessibility of standardized and appropriate treatments for numerous patients (Su et al., 2022).

Despite the robust empirical evidence supporting specific psychological interventions, their extensive implementation in the field remains a significant obstacle (Sadeh-Sharvit et al., 2023). Furthermore, therapists employed in mental health centers are burdened with significant administrative obligations, resulting in an imbalance between work and personal life and the emergence of compassion fatigue in frontline mental health workers. These circumstances challenge the successful implementation of evidence-based practices (Moreno et al., 2020; Lluch et al., 2022; Wilkinson et al., 2017). In light of the complexities surrounding the proficiency of adopting particular psychotherapeutic modalities, patients may experience varying levels of engagement, with some demonstrating resistance to treatment. In such scenarios, therapists must embrace flexible strategies to deliver interventions effectively. Considering the challenges with anxiety disorder management, the integration of innovative technologies such as artificial intelligence (AI) environments and platforms emerges as imperative. These technologies hold the potential to assist therapists in managing and treating patients with anxiety disorders and facilitating precision-based therapeutic approaches while concurrently reducing their workload (Bickman, 2020). Additionally, adopting novel technologies to assess the impact of behavioral interventions and conducting regular evaluations of their efficacy are imperative steps toward enhancing patient outcomes. This necessity highlights the importance of implementing scalable AI-enabled mental healthcare delivery systems capable of facilitating the objective measurement of patient progress, streamlining workflow processes, supporting the customization of treatments, and automating administrative tasks (Sadeh-Sharvit and Hollon, 2020).

The effective delivery of therapy relies on establishing human connection, empathy, and the meticulous consideration of each patient’s needs. While these human elements are invaluable, AI-driven tools can complement therapy by enriching clinicians’ capacity to provide personalized care. AI technologies can support this by executing tasks that traditionally demand human intelligence, such as analyzing complex patterns in patient language, behavior, and emotional expressions. This enhancement enables clinicians to dedicate more attention to the human aspects of the therapeutic relationship, like empathy and comprehension, while harnessing AI to refine treatment approaches, monitor progress, and implement data-inspired modifications to enhance therapy outcomes (Graham et al., 2019). The utilization of AI technologies shows promise from the standpoint of patients as they can be incorporated into mental health treatment to enhance patient accessibility and involvement, ultimately resulting in improved quality of care. AI provides logical predictions and can aid clinicians in interpreting treatment data and enabling data-driven clinical decisions (Kellogg and Sadeh-Sharvit, 2022). Despite these advantages, the widespread utilization of AI-enabled digital decision support systems, tools, and environments in healthcare to effectively manage anxiety disorders and other mental health problems has not been achieved (Bertl et al., 2022; Antoniadi et al., 2021).

To address the gap in current research, this study aims to critically analyze the effectiveness of AI technologies in mental healthcare, particularly in managing anxiety disorders. Although there is ongoing research on the use of AI in mental health, there is a significant lack of understanding regarding the comparative effectiveness of AI-based interventions versus traditional therapeutic methods, specifically concerning personalized care, user engagement, and ethical considerations. This study seeks to fill this gap by exploring several critical research questions, including:

- What is the current state of research on the transformative potential of AI-based interventions in managing anxiety disorders, and how do these interventions compare with traditional therapeutic approaches in terms of efficacy.
- In what ways do AI-based interventions enhance precision and personalization in the treatment of anxiety disorders, particularly through adaptive and data-driven approaches tailored to individual needs?
- How do AI-driven environments, including technologies like VR and chatbots, transform user engagement and treatment adherence in the management of anxiety disorders?
- What are the key ethical, privacy, and regulatory challenges associated with the transformative use of AI technologies in managing anxiety disorders?
- Could the integration of AI-based interventions significantly reduce the reliance on medication for individuals with anxiety disorders, and what are the potential long-term outcomes and implications of such a shift?
- What are the key challenges and opportunities in automating mental healthcare services through AI, and how can these technologies be implemented to maximize their transformative impact in clinical practice?
- What transformative insights can be drawn from recent clinical trials on the efficacy of AI in mental healthcare, and how do these findings inform future research and the development of next-generation AI-driven therapeutic tools?

This review centers on exploring the transformative potential of artificial intelligence (AI) technologies in the management of anxiety disorders, emphasizing their ability to enhance personalized care, improve treatment accessibility, and address current limitations in traditional therapeutic methods.

## 2 Materials and methods

### 2.1 Strategy

A narrative review approach has been chosen for its reliability in exploring specific areas within the subject domain and enabling the synthesis of current theories and concepts (Ferrari, 2015). The aim is to identify the recurring patterns, emerging trends, and gaps in existing literature by adopting a narrative review. The study integrates various advantages of systematic reviews, including search strategies, inclusion and exclusion criteria, study selection, and data extraction. For comprehensive coverage, we have relied on established narrative review frameworks successfully implemented in previous studies (Sianturi et al., 2023; Green et al., 2006; Bourhis, 2017). We performed an extensive literature search across several databases such as Scopus, Web of Science, IEEE Digital Library, ACM, PubMed, Google Scholar, and NIH, covering all the existing literature from the previous decade until the present time. This review provides a comprehensive synthesis of knowledge and findings in the specific field of study and provides valuable insights and perspectives.

The research adopted a thorough approach to conduct a comprehensive literature review. The process began with compiling a comprehensive list of keywords and terms such as “artificial intelligence in mental healthcare,” “AI tools for managing anxiety disorders,” “AI-driven environment for treating anxiety disorders,” “use of AI as therapeutic tools in depression and anxiety,” “customizing anxiety treatment with AI technologies,” “digital AI interventions for anxiety,” “AI in psychotherapeutic approaches to anxiety disorders,” and “managing generalized anxiety disorder with AI-based cognitive tools.” These terms were used to search through several databases and search engines, including Google Scholar, Web of Science, and Scopus. The search was supplemented by a manual review of references within the selected studies to capture all pertinent literature, particularly those studies with concepts interlinked with the field, such as AI’s role in managing anxiety disorders, AI-based platforms for anxiety treatment, AI’s application in psychiatry, AI’s enhancement in mental health management, and the evaluation of psychological factors in anxiety disorders through AI tools. This search methodology enabled the identification of a broad spectrum of research on the application of AI in the treatment of anxiety disorders and broader mental healthcare.

### 2.2 Inclusion and exclusion criteria

The review centered on conducting a comprehensive evaluation of the efficacy of AI environments in managing anxiety disorders. As a result, it was necessary to establish precise inclusion and exclusion criteria to select studies that significantly contribute to this field. The inclusion criteria encompass various essential elements, including studies that prioritize utilizing AI technologies, machine learning algorithms, virtual reality simulation, and chatbots for treating and managing anxiety disorders. The review further covers a range of anxiety disorders, namely generalized anxiety disorder (GAD), social anxiety disorder (SAD), panic disorder, phobias, obsessive-compulsive disorder (OCD), and post-traumatic stress disorder (PTSD). The focus areas in most literature, encompassing interrelated concepts such as depression, anxiety, and other mental health disorders, were also given due consideration.

Moreover, particular attention was given to empirical investigations, such as randomized controlled trials, quasi-experimental designs, and observational studies, as they contribute to compiling comprehensive data concerning the efficacy of AI environments in treating anxiety disorders. Furthermore, studies that are chosen must evaluate outcomes that are relevant to the management of anxiety, including the reduction of anxiety symptoms, improvement in the quality of life, and treatment adherence. The study examined all articles published in the past decade to ensure the literature review is current. Furthermore, including English articles improves the accessibility and practicality of the literature review for a broader range of readers. In order to maintain a comprehensive evaluation of AI-enabled interventions, studies that solely investigate conventional therapies without incorporating AI technologies are excluded. Similarly, research articles that explore mental health conditions other than anxiety disorders are deliberately left out to uphold the review’s specificity. Furthermore, non-empirical sources, including editorials, opinion pieces, conference abstracts, dissertations, and preprints, are excluded from upholding the reliability of the synthesized evidence. A human-centric focus is maintained in the literature review by excluding studies that do not have direct implications for managing anxiety disorders in humans. Moreover, the exclusion of duplicate publications or redundant data from the same study populations during the review process serves to enhance the validity of the findings by preventing data duplication and bias.

### 2.3 Study selection

The selection of studies was conducted in a systematic two-stage process. Initially, titles and abstracts were screened from a comprehensive database search spanning Web of Science, Scopus, PubMed, ACM Digital Library, Google Scholar, IEEE, and PsycINFO. This initial screening aimed to identify studies published between 2014 and 2024, resulting in 560 literature sources. Two reviewers independently assessed these records against predetermined inclusion and exclusion criteria. The criteria specified that the studies be either case studies, randomized controlled trials, or practical experiments focused primarily on assessing the efficacy of artificial intelligence technologies in managing anxiety disorders. These studies needed to detail interventions involving AI technologies with participants assigned to treatment and control groups. Additionally, only studies published in English and available in full text were included, thereby excluding conference papers, preprints, or papers only available in abstract form.

Beyond database searches, the selection process was enriched by consultations with field experts who recommended pivotal studies and unpublished reports. We also conducted hand searches of conference proceedings and scanned the reference lists of identified papers to ensure comprehensive coverage and inclusion of relevant studies. This thorough approach included critical literature that may not have been indexed in the primary databases.

Following the initial screening, 320 studies were selected for a more detailed review. Full-text articles were meticulously examined to confirm relevance and methodological rigor. Discrepancies between the reviewers were resolved through discussion or, if necessary, by consulting a third reviewer.

**Figure 1:**
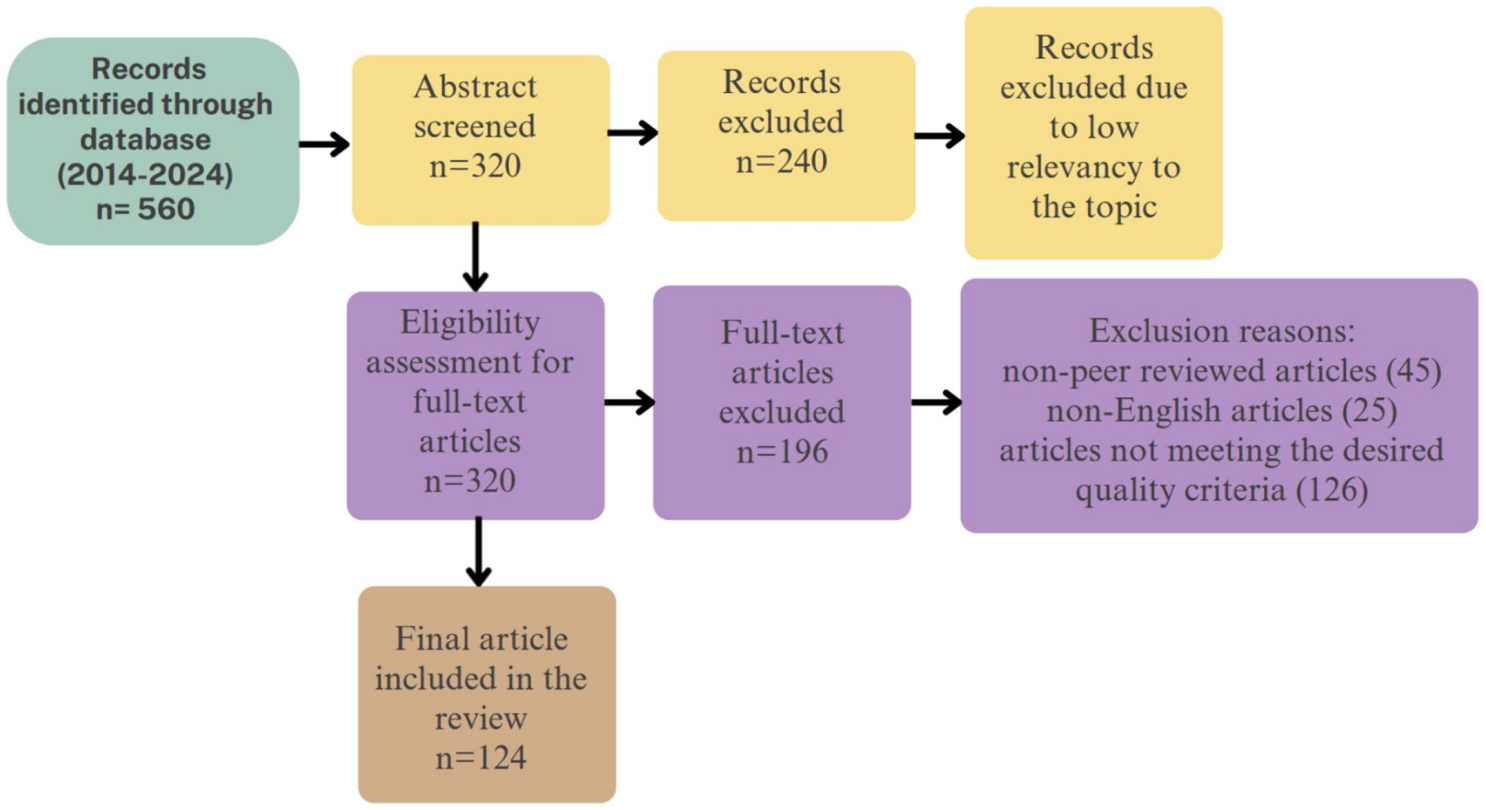
Study selection flowchart.

This detailed examination narrowed the selection down to 196 studies for further analysis. Articles were excluded if they were non-peer-reviewed (Rundo et al., 2020), not in English (Graham et al., 2019), or failed to meet other quality criteria (Kessler et al., 2009). Finally, 125 peer-reviewed articles were thoroughly analyzed to extract insights and evidential support regarding the effectiveness of AI technologies in treating anxiety disorders. Figure 1 depicts the flowchart for the study selection and inclusion process.

### 2.4 Data extraction

The study employed a narrative approach to accommodate the expected study design and results variations. This approach involves qualitatively summarizing the data from the studies included in the analysis.

The study characteristics comprised data on authors, publication year, and study location. This information establishes the framework for the research and its conclusions. Conversely, the study design outlines the research design, encompassing randomized controlled trials or quasi-experimental approaches, and incorporates participant demographics, including gender and age, to enhance the contextual understanding. The sample sizes reflect the number of participants included in the study. A significant focus has been placed on including intervention details, specifically the utilization of AI applications in the study, encompassing the purpose and method of implementation. Furthermore, an examination was conducted on the impact on therapy outcomes and the management of anxiety disorders. Finally, the key findings encompassed the primary outcomes and conclusions from the study on the influence of AI interventions.

### 2.5 Data synthesis

Due to the varying nature of study designs and outcome measures, we used a qualitative narrative framework to synthesize data in this review. This method allowed for a comprehensive examination of the data without the restrictions of meta-analysis techniques, which may not be appropriate when there is high heterogeneity. The narrative synthesis involved a detailed assessment of each included study, focusing on identifying and understanding the diverse impacts of the interventions studied.

The analysis involved finding key patterns, themes, and trends. This included examining how different variables influenced the outcomes and identifying commonalities across studies to draw broader conclusions about the topic. During the analysis, we paid particular attention to contextual and methodological differences that could affect the interpretation of results, such as the settings where AI technologies were implemented and the populations targeted. We integrated the findings to construct a coherent narrative that reflects the potential and limitations of AI tools and environments in managing anxiety disorders. This approach highlights AI technologies’ effects and provides a solid foundation for future research, ensuring that investigations can build on a clear understanding of what has been established previously.

### 2.6 Bias evaluation

The bias evaluation process is critical to uphold the validity and reliability of the findings, addressing potential biases in study selection and data interpretation. A meticulous protocol was designed to mitigate potential biases throughout the review process. Initially, a structured and transparent method was adopted to formulate the research questions and set the inclusion and exclusion criteria, ensuring that the study selection was not influenced by the researcher’s preferences or preconceived notions. Additionally, data extraction was performed using a standard approach to reduce variability and maintain consistency across various studies.

A diverse range of databases was searched to combat publication bias and achieve a comprehensive review, including widely recognized sources. During the data synthesis phase, discrepancies and conflicts were managed by involving an independent reviewer who facilitated critical discussions and consensus building to enhance the credibility and objectivity of the outcomes. This process was not only to eliminate bias but also to emphasize the transparency and reproducibility of the research.

## 3 Findings

Researchers have demonstrated considerable interest and highlighted AI’s potential to enhance anxiety disorder management. AI-enabled interventions are varied and encompass various digital tools, such as AI-enabled virtual reality (VR) environments, chatbots for cognitive behavioral therapy, and machine learning (ML) algorithms designed to customize treatment plans to individual needs. The findings examine the research questions and outline the diverse studies identified throughout the review.

### 3.1 Standard AI techniques for managing anxiety and other mental health disorders

AI has the potential to transform mental health care by adhering to the tenets of personalized care and enhanced accessibility. Existing studies have highlighted the capability of AI technologies to provide continuous support, facilitate early detection of mental health disorders, and reduce the extensive wait times associated with traditional consultations. Furthermore, AI tools enable informed decision-making through robust data analysis, significantly enhancing mental health interventions (Zafar et al., 2024).

Machine learning (ML) has been employed to leverage insights from unstructured data sources, such as clinician notes, to enhance the accuracy of predictions related to anxiety disorders (Thieme et al., 2020). ML techniques enable the development of predictive models that assess the probability of individuals developing mental health disorders by analyzing various clinical factors, including historical data. These predictive outcomes can be used to personalize treatment approaches, recommending specific therapies, medications, or other interventions that align with each individual’s unique characteristics and response patterns (Graham et al., 2019). Furthermore, researchers have also applied ML in classifying mental health conditions using techniques such as support vector machines (SVM), logistic regression, random forests, decision trees, and artificial neural networks (Chung and Teo, 2022). Additionally, there is an emerging pattern of utilizing electronic health records (EHRs) to diagnose mental health disorders by employing ML or hybrid AI techniques. Finally, ML is also being utilized in the development of automated screening tools to aid in the identification of individuals who may be susceptible to specific mental health disorders (Iyortsuun et al., 2023).

Furthermore, ML can be broadly categorized into two main approaches, namely supervised and unsupervised learning. Supervised ML is a technique used to train algorithms using pre-labeled data to categorize or predict outcomes. This training process involves multiple inputs to accurately predict the results, including biological (genetic information), patient medical records, and lifestyle features (Vora et al., 2023). These predictions can take two primary forms: classification, which identifies the type of mental disorder, or quantitative prediction, which indicates the severity of the disease. In contrast, unsupervised learning operates without labeled data and focuses on identifying inherent patterns and relationships within the dataset. This method is critical for analyzing complex datasets like neuroimaging data. Unsupervised learning can aid in discovering new subtypes of mental disorders by identifying these patterns and potentially lead to the development of more effective treatment options based on the acquired information (Thakkar et al., 2024).

Deep learning (DL) is a specialized branch of machine learning that excels at identifying complex patterns and categorizing data. While DL can operate in an unsupervised manner, it is also highly effective in supervised learning tasks where labeled data is used for training. This flexibility has increased interest among researchers aiming to enhance precision in diagnosing and managing health disorders. DL is derived from Artificial Neural Network (ANN) and is inspired by the human brain’s neuronal structure, allowing it to process complex data across multiple layers. These models have become a valuable addition in the context of mental health research, particularly in areas like image classification for neuroimaging data from CT and MRI scans to identify potential markers of mental health conditions (Thakkar et al., 2024).

Reinforcement Learning (RL) involves training algorithms to optimize decision-making abilities by rewarding desired behaviors (Su et al., 2022). This helps the model to adjust treatment plans based on user feedback dynamically. In mental healthcare, the core principle of RL is to assign each trainee a RL agent to assist with personalized tasks or exercises. During the execution of the activities, the RL agents actively interact with the trainee, acquiring knowledge from each distinct interaction utilizing specific RL algorithms and characteristics. This procedure facilitates the development of personalized cognitive systems. The system progressively develops a personalized policy based on the trainee’s continuous performance. The trainee’s performance is a motivating incentive that facilitates effective policy adaptation. The long-term optimization of the trainee’s performance is achieved by carefully adjusting the parameters that determine the difficulty level. Consequently, the system can independently adapt and acquire the optimal approach to enhance cognitive training on an individualized level for each trainee (Zini et al., 2022; Stasolla and Di Gioia, 2023).

Similarly, virtual reality (VR) with AI-integrated techniques has been identified as being used in treating and assessing mental health conditions. VR-based exposure therapy is considered to benefit individuals in managing situations concerning anxiety over a feared situation or context in a safe and controlled manner (Bell et al., 2020). This results in less distress and better therapeutic outcomes. On the other hand, computer vision technology is used to interpret visual data and analyze non-verbal cues, such as facial expressions and gestures, to determine emotional states (Huang et al., 2023). This technology plays a critical role in mental health assessments and can support individuals with neurodevelopmental disorders (Hashemi et al., 2021; de Belen et al., 2020).

AI can transform mental health treatments by providing predictive insights, immediate feedback, and personalized care. This potential for transformation makes adopting these methodologies a significant step toward revolutionizing mental health care.

### 3.2 AI technologies for automating mental healthcare services

There are three primary categories of technologies relevant to mental health management: automation technologies, engagement technologies, and clinical decision support technologies. AI-enabled automation technologies optimize healthcare management processes and service delivery using machine learning and computer vision systems, often leveraging structured data like electronic health records for analysis. Meanwhile, recent advancements indicate the emergence of AI-based engagement technologies, such as Natural Language Processing (NLP), which can be used to develop chatbots and intelligent agents to converse and engage patients using unstructured data like spoken language or text (Bickman, 2020). Additionally, platforms that incorporate NLP and ML can offer chatbot-based screening and assist with routine tasks, which might be neglected due to the various responsibilities of clinicians. Technologies like computer vision, NLP, and ML can perform a wide range of tasks, such as emotion and conversation style recognition, leading to automating initial screening processes. This information can enable the recommendations for treatment or specific types of therapy as per individual needs (Chang et al., 2021). Additionally, AI-based technologies can support automating the data collection process and its analysis before, during, or after sessions, enabling digital surveys and extracting insights from patterns in patient’s speech tone and word choices (Kellogg and Sadeh-Sharvit, 2022).

A critical aspect of AI-enabled screening tools is to improve the accuracy, accessibility, and scalability of the interventions (Smrke et al., 2021). Nonetheless, it is critical to acknowledge that these technologies are specifically developed to assist, rather than substitute, the expertise and know-how of clinicians and help address problems associated with workforce shortage in mental health (Balcombe and De Leo, 2021). Additionally, these tools facilitate the evaluation of patient responses to specific interventions, identifying instances where improvements are insufficient, and prioritizing more intensive care when necessary (Lim et al., 2021). Some key findings from evaluating the automation aspect of AI in mental healthcare settings are detailed in Table 1.

**Table 1:** Key findings from AI for automation in mental healthcare setting.

Technology type	Key applications	Benefits
AI for automation	- Employing computer vision and ML for preliminary assessment and identifying patterns that may suggest symptoms of anxiety, depression, or other mental health issues.	- Enhancing early detection, which is critical for effective intervention.
	- Automated data collection and analysis.	- Increases efficiency and accuracy in patient monitoring.
	- AI-based analysis of patient speech for mood and stress assessment.	- Reducing the administrative workload of clinicians, thereby creating additional time for patient care.
	- Routine task automation, such as scheduling and follow-ups	- Reducing errors and operational costs and improving healthcare delivery.
AI technologies for instant engagement	- Chatbots are designed to provide continuous mental health support and crisis management.	- Providing constant access to support, especially in areas lacking immediate accessibility to mental healthcare services.
	- Interactive applications for behavioral coaching.	- Enhancing patient engagement and adherence to treatment plans.
	- Virtual agents for psychotherapy support through cognitive behavioral therapy and motivational interviewing.	- Enabling extension of therapeutic reach beyond in-person sessions.
AI-based clinical decision support	- Machine learning algorithms for analyzing patient data to predict mental health issues.	- Allows for earlier and more accurate diagnosis, improving outcomes.
	- AI models to customize treatment plans based on individual patient data.	- Personalize treatment and increase its effectiveness by targeting patient-specific needs.
	- Deep learning models to predict patient response to different therapies.	- Optimizing treatment strategies by forecasting which interventions are likely most effective.

### 3.3 Challenges of automating mental healthcare service using AI

While AI-enabled tools and environments offer clear benefits for managing anxiety disorders and other mental health issues, there are notable drawbacks to keep in mind. Implementing AI-based automation in mental healthcare can lead to a dependence on automated systems, resulting in decreased clinician attentiveness. This issue may become acute in complex care situations where automated systems fail to grasp subtle patient needs. Additionally, many AI tools incorporate auto-complete functions that could lead clinicians to overlook errors in medication or therapy names, alerts, or unusual patient responses due to diminished vigilance (Kellogg and Sadeh-Sharvit, 2022; Parasuraman and Manzey, 2010).

Moreover, if AI systems are integrated to perform tasks such as the initial screening or data analysis, clinicians may feel their roles are diminished or their professional importance is undermined (Fiske et al., 2019). Another critical challenge is allocating tasks between AI technologies and clinicians. This uncertainty can affect the adoption and trust in AI technologies, which impacts their effectiveness in clinical settings. Lastly, integrating AI into mental healthcare raises ethical and practical issues. Clinicians and patients may have concerns about data security and privacy, and the potential biases inherent in AI models may lead to poor clinical applicability (Asan and Choudhury, 2021). Additionally, patients may still feel the absence of human elements in healthcare, which further compromises the adoption of AI, especially in critical settings like managing anxiety disorders (Rundo et al., 2020).

### 3.4 Natural language processing and chatbots in mental healthcare setting

NLP is an advanced AI technology that develops tools and environments to facilitate human-like interactions, providing real-time support and psychoeducational content. These chatbots perform dynamic processing of user inputs and deliver responses that simulate therapeutic interactions, enhancing user engagement while offering continuous accessibility-a critical feature in regions underserved by mental health professionals (Ahmed et al., 2023). NLP is currently used for various tasks, from analyzing social media to customer support and monitoring mental health in real-time by evaluating the emotional context in text and speech patterns (Thakkar et al., 2024; Khurana et al., 2023; Zhang et al., 2022). This technology facilitates the development of chatbots that provide instantaneous interaction, immediate support, and valuable insights.

Additionally, chatbots can analyze user inputs in order to identify indications of distress, anxiety, or depression (Bendig et al., 2019). AI chatbots can potentially overcome obstacles in seeking mental healthcare by providing personalized, easily accessible, cost-effective, and stigma-free confidential support. This can facilitate early intervention and generate critical data for research and policy development (Singh, 2023). Another significant benefit is that AI chatbots offer interactions free from human judgment, which is particularly important for individuals hesitant to seek help due to fear of stigmatization or bias (Khawaja and Bélisle-Pipon, 2023). Currently, there are existing evidence-based applications that incorporate chatbots, such as Ada, Wysa, Replika, and Youper, which have been examined in the context of mental health. Although existing research has focused broadly on mental health applications, it has been conducted narrowly on specific disorders such as anxiety. Therefore, a thorough review is essential to assess the variety of features these applications offer (Ahmed et al., 2021).

NLP-based chatbots are gaining traction in mental health interventions. These AI systems are designed for one-on-one interaction, aiming to provide human-like responses and support. A common data source for these chatbots comes from user interactions within dedicated mental health apps, such as mindfulness training apps or chatbots specifically designed for anxiety or depression management (Zhang et al., 2022). In mental healthcare, AI-driven tools with NLP and ML can be integrated to evaluate data for emerging trends in anxiety disorders, risk elements, and potential solutions (Zafar et al., 2024; Straw and Callison-Burch, 2020). However, a recent study emphasized the need for AI chatbots to incorporate human values such as empathy and ethical considerations, as this integration can address the limitations of AI systems and improve their interaction with users, making them more effective and trustworthy in sensitive domains like mental health (Balcombe, 2023). Additionally, the study highlights the pressing need for guidelines and regulations to ensure that AI chatbots are developed and deployed responsibly by incorporating principles of fairness, accountability, and transparency in AI design and operation (Timmons et al., 2023; Joyce et al., 2023). Furthermore, it has been indicated that education and training regarding the capabilities and limitations of AI chatbots are vital for both users and providers as they can help mitigate unrealistic expectations and promote effective use of technology in mental healthcare (Balcombe, 2023).

**Figure 2:**
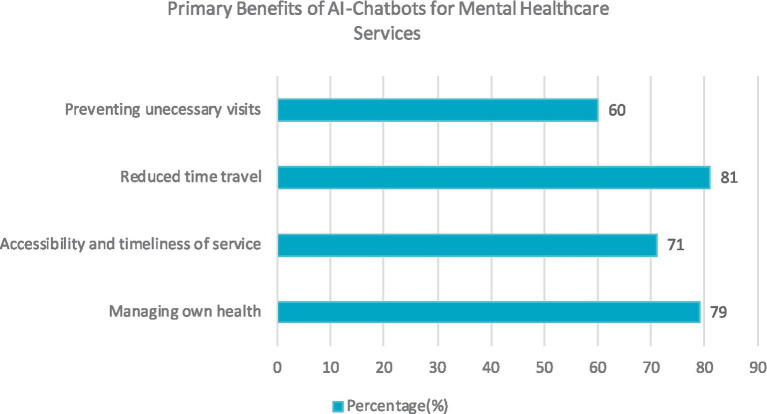
Primary benefits of AI-chatbots for mental healthcare services.

An investigation conducted by Mulvenna et al. examined the viewpoints of mental health professionals regarding integrating chatbots as a means of supporting mental health services. The majority of participants recognized the advantages of AI-enabled chatbots in mental healthcare, with some expressing the potential for chatbots to assist in their mental health management. Nonetheless, a prevailing concern was the perceived deficiency of chatbots in comprehending or expressing human emotions. However, as professionals acquired more experience, their confidence in the efficacy of healthcare chatbots in aiding patient self-management grew (Sweeney et al., 2021). The primary benefits of chatbots for mental healthcare service delivery are highlighted in Figure 2.

**Figure 3:**
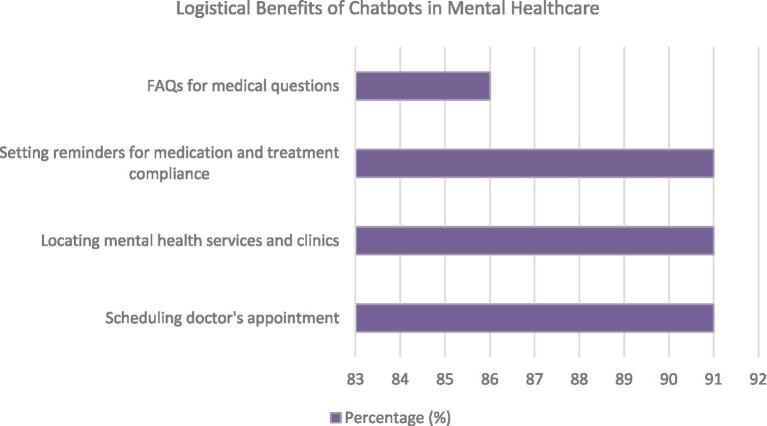
Logistical benefits of chatbots in mental healthcare.

The study conducted by Mulvenna et al. also highlighted the potential logistical advantages of incorporating chatbots into mental healthcare. These benefits can potentially enhance mental. Figure 3 displays the mentioned benefits.

**Figure 4:**
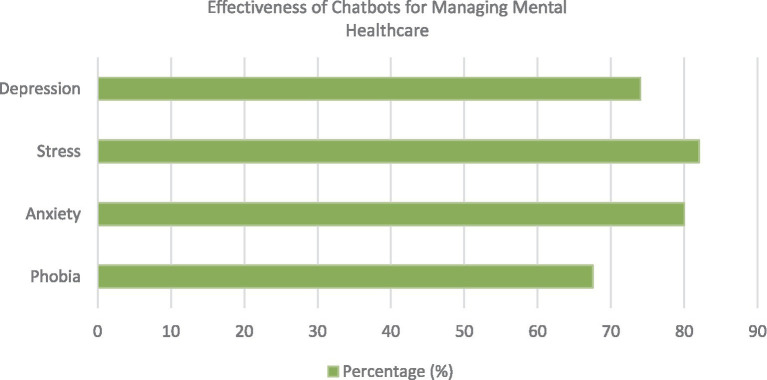
Logistical benefits of chatbots in mental healthcare.

Moreover, chatbots are seen as potentially valuable tools in the domains of self-management (39%), education (36%), and training (35%). These tools are additionally recognized as beneficial aids in cognitive behavioral therapy, with nearly a quarter of professionals affirming their capacity to provide continuous support with minimal user involvement (Sweeney et al., 2021). The respondents indicated that they felt chatbots could effectively manage several mental health conditions, as depicted in Figure 4.

Notwithstanding these advantages, obstacles to the implementation of mental healthcare chatbots exist. A notable percentage of the participants (54%) emphasized multiple concerns, such as the chatbots’ limited understanding or expression of human emotions (86%), inadequate provision of comprehensive care (77%), and insufficient intelligence to assess clients (73%) accurately. Moreover, a considerable percentage of the participants (59%) raised concerns regarding protecting data privacy and confidentiality (Sweeney et al., 2021).

### 3.5 Wearables for managing anxiety disorders

A recent systematic review and meta-analysis assessed the efficacy of wearable artificial intelligence (AI) technologies in detecting and predicting anxiety disorders, indicating promising yet suboptimal performance. The meta-analysis included 21 studies, yielding a pooled mean accuracy of 0.82, alongside sensitivity and specificity values of 0.79 and 0.92, respectively. These findings suggest that wearable AI can reliably identify the presence and absence of anxiety in most instances. Nevertheless, due to the current performance limitations, wearable AI is not advised for exclusive clinical application. Instead, integration with conventional diagnostic methods is recommended to enhance accuracy. Furthermore, the study encourages the inclusion of additional data sources, such as neuroimaging, to improve the diagnostic capabilities of wearable technologies (Abd-alrazaq et al., 2023).

There are numerous wearable devices that incorporate machine learning (ML) algorithms to process and analyze the data they collect, providing users with valuable insights and real-time feedback on their health and wellness. This enables continuous health monitoring and management (Nahavandi et al., 2022). Recent research by Gedam and Paul has explored the application of ML algorithms to detect stress using data from multiple wearable sensors. This study highlighted heart rate and galvanic skin response as the most effective sensors (Gedam and Paul, 2021). Moreover, researchers like Cruz et al. have emphasized the importance of detecting and predicting Panic Disorder (PD), utilizing anomaly detection algorithms to distinguish between panic and non-panic states (Cruz et al., 2015). The existing literature regarding the application of machine learning in the analysis of physiological data for mental health monitoring through wearable technology is growing, although it remains in its early stages (Gomes et al., 2023). The findings concerning the most effective AI models and other features employed in using wearable sensors and hybrid approaches for anxiety and other mental health disorder detection and monitoring are summarized in Table 2.

**Table 2:** Commonly used AI models and algorithms and key features for anxiety detection and mental health monitoring.

Features	References
Supervised machine learning algorithms	
Decision tree	Klos et al. (2021) and Anmella et al. (2023)
Random forest (RF)	Plana et al. (2022) and Koutsouleris et al. (2022)
Support vector machine (SVM)	Sadeh-Sharvit et al. (2023), Xie et al. (2022), Ancillon et al. (2022), Danieli et al. (2022), and Su et al. (2022)
K-nearest neighbor (KNN)	Sabour et al. (2023), Yao et al. (2023), Viduani et al. (2023), Kaywan et al. (2023), and Manocha et al. (2019)
Logistic regression	(Opoku Asare et al., 2021)
AdaBoost	Cruz et al. (2015) and Gomes et al. (2023)
XGBoost	Fulmer et al. (2018)
Unsupervised machine learning algorithms	
Bayesian networks	Monteith et al. (2022), Cuthbert and Insel (2013), and Shatte et al. (2019)
Artificial neural network (ANN)	Goel et al. (2010), Satiani et al. (2018), Olawade et al. (2024), and Rosenfeld et al. (2021)
Long short-term memory (LSTM)	Abdullah et al. (2016)
Convolutional neural network (CNN)	Linardatos et al. (2021), Carr (2020), Markus et al. (2021), Amann et al. (2020), Lipton (2018), and Rudin (2019)
Biomarkers as input for ai algorithms for anxiety and stress detection	
Heart rate	NA
Galvanic skin response	NA
ECG	NA
Physical activity	NA
Electrodermal activity	NA
EEG	NA
Behavioral	NA
Skin temperature	NA
Scales used	
Self-Reported Anxiety Scale (SAS)	NA
Self-Reported Depression Scale (SDS)	NA
Ground truth assessments	
State–Trait Anxiety Inventory (STAI)	NA
Depression and Anxiety Mood Scale (DAMS)	NA
Observations	NA
Interviews	NA
Labeling	
Hamilton Anxiety Rating Scale	NA
Self-help for anxiety management	NA
Validation	
Leave-one-out-cross validation	NA
10-fold cross validation	NA
K-fold cross-validation	NA
Hold-out cross-validation	NA

Table 2 presents an extensive overview of the commonly used AI algorithms for anxiety detection and mental health monitoring. This includes Support Vector Machine (SVM), Random Forest (RF), AdaBoost, Decision Trees, Artificial Neural Networks (ANN), K-nearest Neighbors (KNN), Bayesian Networks, Logistic Regression, Long Short-Term Memory (LSTM), XGBoost, and Convolutional Neural Networks (CNN). These algorithms are referenced across various studies, demonstrating their efficacy and application in interpreting complex physiological and behavioral data for mental health assessment.

Key biomarkers such as Heart Rate, Galvanic Skin Response, ECG, Physical Activity, and others are used as inputs to enhance the precision of the models. The validation of these algorithms is conducted using established scales like the State–Trait Anxiety Inventory (STAI) and Self-Reported Anxiety Scale (SAS), which help ascertain their accuracy and reliability in clinical settings. This integration of advanced computational methods and multiple biomarkers underscores the evolving landscape of mental health diagnostics and holds the potential to improve personalized treatment plans and real-time monitoring of mental health conditions.

### 3.6 Outcomes of randomized trials and practical experiments

To understand the efficacy of AI in managing anxiety disorder and other interconnected mental health disorders, we have examined various randomized controlled trials, pilot studies, and practical implementations. In recent years, the integration of AI into mental health interventions has garnered significant attention, resulting in novel approaches to treatment and mental healthcare service delivery, with an emphasis on improving accessibility and effectiveness. The discussion is aimed not only at assessing the measurable outcomes of such trials but also at understanding the broader implications of integrating AI into routine clinical practice for anxiety and other mental health disorders. The outcomes are summarized in Table 3.

**Table 3:** Summary of randomized trials and practical implementations.

Authors	Methods implemented	Measures	Intervention	Key findings	Participants	Outcomes	Limitations
Klos et al. (2021)	8-week interaction with Tess chatbot, control group with psychoeducation book	Depression and anxiety symptoms	AI Chatbot “Tess”	High engagement with Tess; no significant intergroup effects but intragroup anxiety reduction noted	181 Argentinian university students	Demonstrated Tess’s potential as a supportive tool	Lack of significant intergroup effects
Anmella et al. (2023)	Reliability testing, simulation study, and one-month interaction using self-assessment tools	Burnout, anxiety, depression scores	Vickybot Chatbot	No significant changes in anxiety or depression; moderate reduction in burnout	34 PC visitors and healthcare workers	Effective in identifying suicide risks and reducing burnout	Small sample size; short duration of study
Sabour et al. (2023)	Randomized control trial with interventions featuring cognitive and emotional support	Depression (PHQ-9), Anxiety (GAD-7), Insomnia Severity Index	AI Chatbot “Emohaa”	Significant improvements in depression, anxiety, and insomnia	301 participants in mainland China	Validated the efficacy of Emohaa in reducing mental distress	Limited cultural and demographic diversity in participant pool
Yao et al. (2023)	Development and evaluation of three models; rule-based and generative models tested for empathy and contextuality	User satisfaction and empathy for postpartum mood and anxiety disorders	Three different chatbot models	Rule-based model preferred for higher empathy and contextual appropriateness	Caregivers with postpartum mood and anxiety disorders	Highlighted rule-based chatbots as effective	Generative model sometimes produced confusing responses
Viduani et al. (2023)	WhatsApp-based chatbot administering structured questionnaires; pilot testing with feedback integration	Mood assessments via Short mood and feelings questionnaire (SMFQ)	WhatsApp-based IDEAbot	High engagement and compliance; effective for ongoing mood assessments	Adolescents participating in the IDEA-RiSCo study	Demonstrated high efficiency and acceptance among adolescents	Small initial testing phase; limited long-term data
Kaywan et al. (2023)	Development using Dialogflow; mixed-methods approach with participant surveys	Depression detection	DEPRA Chatbot (non-clinical trial)	Successful early depression detection among participants	50 participants across Australia	Showed potential as a mass-screening tool for depression	Limited participant diversity; primarily non-clinical sample
Manocha et al. (2019)	Implementation of a Weighted-Naïve Bayes classifier and ANFIS-GA for health monitoring	GAD prediction and monitoring	Cognitive-intelligence in a AI-assisted fog-cloud architecture	Effective in predicting and monitoring health adversities related to GAD	15 elderly individuals	Improved accuracy and speed in health monitoring	Small sample size; specific to elderly demographic
Fulmer et al. (2018)	Randomized into groups with varying access durations to Tess; surveys measured depression and anxiety	Depression (PHQ-9), GAD-7	AI Chatbot Tess	Significant reductions in depression and anxiety symptoms in intervention groups	75 college students from 15 universities	Tess proved effective in alleviating symptoms	Short intervention durations; potential bias in self-reporting
Sadeh-Sharvit et al. (2023)	Use of an AI platform to assist therapists in outpatient CBT; comparison of treatment outcomes and operational efficiency	Anxiety and depression symptom reduction	AI platform developed by Eleos Health	Improved treatment outcomes and operational efficiency in the AI-supported group	47 adult participants in a community-based clinic setting	Enhanced clinical outcomes and efficiency	Small sample size; limited generalization to other settings
Xie et al. (2022)	Data collection involving facial expressions and movements; integration of scale scores with video data	Diagnostic accuracy of depression and anxiety	CNN and LSTM model analysis	High accuracy in diagnosing depression and anxiety	303 diagnosed subjects	Advanced diagnostic performance	Focus on diagnosed individuals limits broader applicability
Ancillon et al. (2022)	Review of studies using biosignals and machine learning models; emphasis on feature selection and algorithm performance	Anxiety detection accuracy	Machine learning models analyzing biosignals	High accuracy in anxiety detection using combined biosignals	Studies with 10 to 102 participants	Showcased the potential of machine learning in anxiety detection	Variability in study designs and sample sizes
Danieli et al. (2022)	8-week RCT comparing CBT delivered remotely with groups receiving various combinations of CBT and mHealth support	Anxiety and stress levels	CBT with AI-enhanced mHealth Agent	No significant differences between groups, but noted benefits of AI integration	45 active workers aged over 55 years	Indicated effective integration of AI in therapeutic processes	Lack of long-term sustainability of treatment effects
Su et al. (2022)	Multicenter RCT comparing AI-assisted psychotherapy plus medication against medication alone; assessments with the Hamilton Anxiety Rating Scale and other scales	Hamilton Anxiety Rating Scale (primary), early treatment improvement	AI psychotherapy robot combined with medication	Protocol for assessing the efficacy of AI-assisted psychotherapy combined with medication	708 patients with anxiety disorders recruited from eight hospitals	Intends to improve access and effectiveness of treatments	Study still in recruitment phase, no results available

The table offers valuable insights into the diverse applications and outcomes of AI and ML in mental healthcare. While some studies, like the evaluation of the “Emohaa” chatbot in China, demonstrated notable efficacy in managing mental health symptoms, others, such as the Vickybot trial, highlighted the variability in AI’s effectiveness, particularly in altering core symptoms like anxiety and depression, despite showing promise in reducing burnout among healthcare workers. The findings of the various randomized controlled trials (RCTs) indicate that a more inclusive research approach is needed in future trials to ensure that findings are applicable across diverse populations. This is critical as the geographical concentration of these trials in high-resource settings may limit their applicability in lower-resource environments. In this context, a study by Plana et al. (2022) highlighted significant methodological shortcomings in medical machine learning RCTs, such as a high risk of bias and poor demographic inclusivity, which could affect the generalizability of the results.

While the studies collectively suggest that AI and ML have the potential to revolutionize mental healthcare treatment outcomes and patient care, the actual implementation in clinical settings is contingent on overcoming significant challenges related to trial design, data integrity, and ethical reporting standards. These insights are critical for understanding the current landscape of AI in healthcare and underscore the need for methodological rigor, enhanced reporting transparency, and greater inclusivity in future research designs. This synthesis serves as a valuable resource for stakeholders in healthcare and technology, guiding the refinement and implementation of AI-driven interventions in clinical settings.

## 4 Discussion

The integration of AI into mental healthcare represents a significant transformation in managing conditions such as anxiety disorders and offering advanced capabilities for diagnosis, personalizing treatment options, and broader accessibility. However, there are significant challenges in applying these technologies in real-world clinical settings. While AI can enhance predictive accuracy, there remains a notable gap between AI-generated results and the practical demands of healthcare environments, particularly in mental healthcare service delivery (Koutsouleris et al., 2022). Research indicates that while machine learning algorithms have shown promise in mental health applications, some studies suggest that their improvements over existing models might not be substantial enough to alter clinical decision-making processes significantly (Goel et al., 2010). Therefore, there is a critical need to refine AI models to bridge this gap so that they align more closely with clinical evaluations. This alignment is essential to expand access to diagnostic and prognostic services, particularly during crises or emergencies (Satiani et al., 2018).

The incorporation of AI can also enhance mental health service delivery by performing routine screenings, telehealth services, and personalized therapy, which contributes to the accessibility and efficacy of treatment (Olawade et al., 2024). A key benefit of AI is its ability to analyze vast amounts of data, identifying patterns that may be difficult for clinicians to detect manually. This comprehensive view of a patient’s mental health can help devise personalized treatment plans, potentially benefiting those reluctant to seek help due to stigma (Rosenfeld et al., 2021). AI can also support continuous symptom monitoring and early relapse detection, allowing clinicians to focus more on therapeutic relationships and quality care (Monteith et al., 2022).

The development of AI systems for specific tasks in a mental healthcare setting can enhance intervention strategies and therapy recommendations. In this context, the concept of “weak AI” may be suitable as it is more controllable and better suited for mental health treatment needs (Koutsouleris et al., 2022; Cuthbert and Insel, 2013). While some fear that AI could replace human expertise, others believe that healthcare professionals will continue to play a critical role; a collaborative approach with human expertise and AI can improve patient care. This collaboration could lead to enhanced patient management, where AI assists in identifying symptoms and supporting evidence-based decision-making (Koutsouleris et al., 2022). The potential of AI in diagnosing and treating mental health conditions such as anxiety and depression is immense (Shatte et al., 2019). In general, traditional diagnostic methods, which often rely on self-reporting and clinician assessments, are plagued by challenges such as stigma, social desirability bias, and a general tendency toward under-diagnosis (Opoku Asare et al., 2021). However, AI can mitigate these challenges by using vast behavioral and linguistic data to identify subtle indicators of mental health issues. AI algorithms can diagnose conditions early and more accurately, thereby preventing symptom escalation and reducing the burden on healthcare systems (Zafar et al., 2024; Abdullah et al., 2016).

However, implementing AI in mental health care necessitates careful consideration of ethical and practical challenges. AI and ML models, by nature, introduce a degree of uncertainty that must be managed when they fail to perform as expected in real-world scenarios. This indicates the importance of evaluating AI technologies not solely based on their predictive capabilities but also their practical applications, considering the tradeoffs between model simplicity, speed, explainability, and accuracy (Linardatos et al., 2021; Carr, 2020). Explainability is essential in clinical settings as it enables transparency in AI-driven decisions, especially when these decisions affect patient outcomes (Markus et al., 2021). In general, explainability can be an inherent feature of simpler models, such as linear or logistic regression, or approximated in more complex models like artificial neural networks (ANNs). Inherent explainability offers more accurate insights since it directly reflects the model’s decision-making process. In contrast, approximated methods are designed to make sense of “black-box” models like ANNs, where millions of internal parameters may make interpretation challenging (Amann et al., 2020). However, this introduces a trade-off between the high performance of advanced models and their interpretability, posing a significant challenge for the development of clinical decision support systems. As Lipton (Lipton, 2018) observes, the opacity of black-box models makes it difficult for stakeholders to comprehend the reasoning behind AI predictions, undermining confidence in these systems. Moreover, Rudin (2019) highlights that models with higher explainability provide transparent reasoning processes, which is critical when clinicians must justify or communicate AI-driven decisions to patients. Based on the findings from these studies, it is evident that balancing key factors such as model simplicity (which enables quicker deployment), speed (crucial for real-time applications), and accuracy (essential for precise predictions) is critical to improving the usability and acceptance of AI in mental health care.

Furthermore, AI’s impersonal nature can overlook the nuance of emotional aspects crucial in mental health assessment (Li et al., 2023). This challenge is compounded by concerns highlighted across research regarding data security, informed consent, confidentiality, trust, and the management of cognitive biases, all of which are particularly significant in healthcare applications (Thakkar et al., 2024; Rigby, 2019; Lovejoy et al., 2019; McCradden et al., 2023). While AI has made strides in many areas, its ability to consistently account for the emotional subtleties present in human interactions remains limited. There are also concerns regarding algorithmic biases, as AI systems are only as objective as the data they are trained on. In this case, it has been identified that biases may be mitigated through mixed-initiative interfaces that enhance shared decision-making (Bickman, 2020; Lattie et al., 2020). Additionally, the accuracy of AI tools, including wearables, can vary, raising concerns about their reliability in real-world applications. Acceptability also fluctuates across cultural and geographic boundaries, with some communities expressing hesitance toward machine-led care due to cultural perceptions and trust issues (Zafar et al., 2024). The limited availability of technology in resource-limited settings is another concern highlighted in various research, as it may create a digital divide in mental healthcare access (Nilsen et al., 2022).

Despite the concerns, realizing the potential of AI requires a considered and ethical approach to its implementation, as well as addressing issues such as data privacy, the need for clinician training on AI insights, and the broader ethical implications of technology in healthcare. The advancement of mental healthcare requires a collaborative approach, wherein AI complements the knowledge and skills of healthcare professionals, ultimately enhancing human capabilities instead of replacing them. By promoting a symbiotic relationship between human empathy and the analytical capabilities of AI, the mental health sector can utilize these technologies to propel the quality and reach of care, ultimately leading to a substantial enhancement in assistance for individuals with mental health conditions.

### 4.1 Strategic recommendations for implementing AI in mental healthcare

1. Development of mixed-initiative interfaces: Integrating AI systems that assist clinicians by delivering comprehensive, real-time data analysis while still upholding their authority in the final decision-making process. The incorporation of personalized patient data into broader clinical knowledge is anticipated to improve shared decision-making.
2. Addressing training and skill acquisition: The implementation of educational programs and ongoing training is necessary to facilitate clinicians’ comprehension and proficient utilization of AI tools. The educational program should emphasize AI’s capacities and constraints, equipping healthcare professionals with the knowledge to use these tools responsibly and efficiently.
3. Regulatory and ethical frameworks: The implementation of strict regulations and ethical guidelines is necessary to oversee the application of AI in mental healthcare. These frameworks aim to ensure that AI tools adhere to patient autonomy, confidentiality, and unbiased decision-making, thereby safeguarding clinical outcomes. Developers and healthcare institutions must align AI tools with existing regulatory standards such as the General Data Protection Regulation (GDPR) in Europe, which governs data privacy and security (GDPR, 2016). Additionally, the U.S. Food and Drug Administration (FDA) has provided guidelines for software as a medical device (SaMD), emphasizing the importance of transparency, validation, and accuracy of AI tools before clinical implementation (Center for Devices and Radiological Health, 2017).
From an ethical perspective, frameworks such as fairness, accountability, and transparency (FAT) should guide AI development to prevent reinforcing biases in AI-driven systems (Barocas et al., 2024). Furthermore, AI developers need to ensure informed consent, uphold patient confidentiality, and address potential algorithmic bias to build trust among users and clinicians (Mittelstadt et al., 2016). Ethical guidelines from the World Health Organization (WHO) concerning digital health and AI ethics offer additional direction, helping ensure that AI-based interventions align with global public health priorities and human rights standards (World Health Organization, 2021). Hence, incorporating these regulatory and ethical frameworks will be vital to creating AI systems that are both effective and socially responsible.
4. Enhancing patient-clinician relationships: Utilize AI technology to enhance, rather than replace, the therapeutic alliance between patients and clinicians. The role of AI should be perceived as a means to enhance this association, offering well-informed and empathetic aid, thus fortifying the essential trust necessary for effective mental healthcare.

## 5 Conclusion

Despite the notable benefits of AI in mental healthcare, such as improved accessibility, personalized treatment, and enhanced operational efficiency, several challenges must be addressed, including privacy concerns, potential biases, and the necessity of preserving a human touch in delivering care. Achieving a balance between these advantages and disadvantages necessitates the establishment of strong ethical frameworks, ongoing monitoring and enhancement of AI systems, and a concerted effort to integrate AI tools in a manner that complements rather than supplants human care. The objective of future research and policy development should be to tackle these challenges, ensuring that AI contributes positively to mental health outcomes while maintaining the highest standards of care and safeguarding patient privacy and dignity. The effective incorporation of AI into mental healthcare will rely on our capacity to navigate these complexities, establishing an environment where technology and human expertise work together to enhance service delivery and patient care.
